# Non-Alcoholic Fatty Liver Disease Among Newly Diagnosed Type 2 Diabetes Mellitus in North Bihar and Missed Opportunities for Early Diagnosis

**DOI:** 10.7759/cureus.75661

**Published:** 2024-12-13

**Authors:** Supriyo Mukherjee, Bishnupriya Mukherjee, Anjali Goswami, Chun Shing Kwok, Anne Phillips

**Affiliations:** 1 Department of Medicine, Dr Mukherjee S Clinic Pvt. Ltd. and Research Centre for Diabetes Hypertension and Obesity (RCDHO), Samastipur, IND; 2 Department of Medicine, Research Centre for Diabetes Hypertension and Obesity (RCDHO), Samastipur, IND; 3 Cardiology, University Hospitals of North Midlands National Health Service (NHS) Trust, Staffordshire, GBR; 4 Faculty of Health Education and Life Sciences, Post-Qualifying Healthcare Practice, Birmingham City University, Birmingham, GBR

**Keywords:** diagnosis delay, missed opportunities, non-alcoholic fatty liver disease, predictors, prevalence, type 2 diabetes mellitus

## Abstract

Background: There are no studies investigating missed opportunities for earlier diagnosis in newly/recently detected Type 2 Diabetes Mellitus and Non-alcoholic Fatty Liver Disease in the region of Bihar, India.

Methods: This study is a single-center cross-sectional study undertaken at the Research Centre for Diabetes Hypertension and Obesity, Samastipur, Bihar, India. The study collected data from newly/recently diagnosed persons with T2DM. The study was conducted between December 2022 and May 2023.

Results: A total of 148 people with newly diagnosed T2DM were included (median age 47, 46.6% female), and 109 patients with liver disease on ultrasound evaluation. The majority of the persons detected with diabetes were symptomatic. The commonest group of typical symptoms were excessive hunger, urinary frequency, excessive thirst, and evening fatigue, which were present in 46 individuals with liver disease. The best pathway includes a group of persons who visited specialists and MBBS doctors once they felt their symptoms should be evaluated by them and diagnosed within two months of their symptom onset. Unfavorable pathways causing delays in diagnosis and hindering efficient care involve individuals with diabetes seeking help from village doctors, pharmacists, and Aayush doctors, thereby contributing to missed opportunities.

Conclusion: NAFLD is prevalent in new T2DM, especially among those with high fat intake and obesity markers. This study could initiate future research aiming to improve NAFLD management and decrease associated complications in newly diagnosed persons with T2DM.

## Introduction

Effective diabetes management relies on timely diagnosis and intervention. In India, 101 million individuals are diagnosed with diabetes, and an additional 136 million have prediabetes [1]. Previous studies showed that the majority of Indians (44.48%) have diabetes with non-alcoholic fatty liver disease (NAFLD), showcasing a higher prevalence among males (58.64%) than females (36.91%). Recently, a study published estimated the prevalence of NAFLD in patients with newly diagnosed T2DM. Ultrasound evaluations revealed liver disease in 109 patients, with females showing a preponderance. The study highlighted the high prevalence of NAFLD in newly diagnosed T2DM patients, particularly among those with obesity-related markers or high-fat consumption [2].

This study investigates the intricate landscape of Type 2 Diabetes Mellitus (T2DM) in North Bihar. A complex interplay of genetic predisposition, environmental influences, and lifestyle choices heightens the risk for specific ethnic groups [3]. While T2DM can be diagnosed through tests like HbA1c or fasting plasma glucose levels, detecting asymptomatic cases is challenging. Symptoms like blurred vision and frequent urination can be ambiguous, leading to misdiagnosis or delayed diagnosis [4]. Access to diagnostics, referrals, specialist assessments, and cost-effective interventions can improve outcomes [5].

However, there are often missed opportunities in T2DM care, worsening the condition and increasing complications, especially with poor risk factor management [6]. This affects individuals and society, increasing the healthcare burden [7]. Understanding the pathways to diagnosis and patient experiences can help identify barriers and improve early detection [8]. This study aims to assess the most effective care pathway by examining patient decision-making processes for newly diagnosed T2DM and non-alcoholic fatty liver disease, aiming to identify missed opportunities for earlier diagnosis and potential harm reduction.

Research aim

This study aims to investigate missed opportunities for earlier diagnosis in newly diagnosed type 2 diabetes mellitus (T2DM) and non-alcoholic fatty liver disease (NAFLD) in North Bihar, India.

Research hypothesis

It is hypothesized that there are significant missed opportunities for early diagnosis of T2DM and NAFLD in North Bihar, particularly delays in seeking appropriate HCPs and referrals, leading to complications and hindering effective management strategies.

## Materials and methods

This study is reported under the recommendations of the STROBE criteria.

Ethical considerations

This study was conducted under the Declaration of Helsinki and the Indian Council of Medical Research. Ethical approval was obtained from the Gurushree Hi-Tech Multi Specialty Hospital ethics committee (8/22/004IEC) in Bangalore, India, and the Faculty Academic Ethics Committee in the Health, Education, and Life Sciences at Birmingham City University, U.K. (Mukherjee /#10953 /sub2 /R(C) /2022 /Nov /HELS FAEC).

Study design and setting

This single-center cross-sectional study was undertaken at the Research Centre for Diabetes Hypertension and Obesity (RCDHO) in Samastipur, Bihar, India. As a private welfare clinic, RCDHO is an outpatient healthcare provider that addresses the needs of over 20,000 patients with Type 2 Diabetes, catering mainly to resource-constrained individuals by detecting, treating, and facilitating referrals to appropriate sub-specialists [9].

Study population

The study collected data from individuals newly diagnosed with T2DM or diagnosed within six months from December 15, 2022, to May 31, 2023. The study inclusion criteria were male or female patients aged 18 years or older who had a new diagnosis of T2DM or were recently diagnosed within six months and consented to participate in the study. Data were collected on the study's sociodemographic and clinical parameters required for patients who provided written informed consent. Patients were excluded from the study if they had a diagnosis of type 1 diabetes mellitus, consumed alcohol regularly, were pregnant or breastfeeding, had a history of traditional medicine use, had a history of acute hepatitis, chronic liver disease, and malignancy that might involve the liver. Women of childbearing age underwent urine tests for pregnancy, and those who had a negative pregnancy test and met the inclusion criteria were invited to take part in the study.

Data collection

The case report form utilized in this study gathered detailed information on demographic data, comorbidities, risk factors, and anthropometric measurements such as height, weight, waist and hip circumferences, and waist-to-hip ratio. (See Supplementary file). Type 2 diabetes (T2DM) was diagnosed according to WHO guidelines, which include fasting plasma glucose, HbA1c, and random blood glucose levels. Blood tests performed included fasting blood glucose, HbA1c, alanine aminotransferase, and lipid profile. An ultrasound examination, carried out by a blinded sonographer, was used to evaluate the presence and severity of fatty liver disease.

Part B of the data collection tool recorded a detailed history of symptoms, from their onset to the first healthcare visit. Symptoms were classified as typical (e.g., excessive hunger and thirst) or atypical (e.g., light-headedness and lethargy). Each step in the process of symptom perception and healthcare-seeking behavior was carefully documented. For diagnosing non-alcoholic fatty liver disease (NAFLD), ultrasound criteria were based on liver echotexture, vascular blurring, and narrowing of hepatic veins. Grading for NAFLD severity was as follows: Grade I: Mild diffuse hyper-echogenicity. Grade II: Moderate hyper-echogenicity with impaired intrahepatic vessel visibility. Grade III: Severe hyper-echogenicity with diaphragm and posterior liver obscuration.

Study outcomes

Primary outcome measure: Assessing non-alcoholic fatty liver disease prevalence among newly diagnosed T2DM individuals in North Bihar, India.

Secondary outcome measure: Investigating symptom development and clinical presentation in newly diagnosed individuals with T2DM and NAFLD to identify potential missed opportunities in earlier diagnosis.

Statistical analysis

Data from case report forms were put in Microsoft Excel, and statistical analysis was undertaken on Stata version 13.0 (College Station, TX) and IBM Corp. Released 2021. IBM SPSS Statistics for Windows, Version 28.0. Armonk, NY: IBM Corp. Descriptive statistics were presented for the collected variables for the overall cohort and stratified according to the presence or absence of NAFLD. For continuous variables that were normally distributed, the mean and standard deviations were presented; for those that were not normally distributed, the median and interquartile range were reported. For categorical variables, the number of patients and the percentages were presented. To test for statistical differences in characteristics between the group with NAFLD and those without NAFLD, the t-test (for normally distributed continuous data), median test (on Stata for data that was not normally distributed and continuous data), and Chi2 test were used (for categorical variables).

## Results

Symptom onset, presentation, and progression and its relation to liver disease

Out of 148 individuals newly diagnosed with type 2 diabetes mellitus (T2DM), 109 (73.7%) presented with liver disease, and 39 (26.3%) did not. The proportion of men and women with liver disease was almost equal: 51 (46.8%) male and 58 (53.2%) female. However, among those without liver disease, 28 (71.8%) were male, and 11 (28.2%) were female, suggesting gender-specific differences in liver disease prevalence. The prevalence of non-alcoholic fatty liver disease (NAFLD) in the T2DM population emphasizes the importance of liver disease screening in these patients. The association was statistically significant, with a p-value of 0.0124. A chi-square test of independence revealed a significant association between gender and liver disease presence, χ² (1) = 6.25, p < 0.05. This indicates that liver disease prevalence differs significantly between males and females in the studied population (Table 1).

**Table 1:** Distribution of newly/recently detected persons living with Type 2 Diabetes Mellitus (T2DM) based on the presence or absence of liver disease χ²: Chi-square value

	Liver Disease Present	Liver Disease Absent	χ²	p-value
Total	109	39	6.247	p < 0.05
Male	51 (46.8%)	28 (71.8%)	-	-
Female	58 (53.2%)	11 (28.2%)	-	-

Among the 109 individuals with liver disease, 73 (66.9%) had typical symptoms (e.g., thirst, dry mouth, nocturia, polyuria, weight loss), while 32 (29.4%) presented with atypical symptoms (e.g., nocturnal foot cramps, lethargy, light-headedness), and 4 (3.7%) were asymptomatic. The majority of those with typical symptoms had grade I or II liver disease, while those with atypical symptoms were more likely to have grade II liver disease. A small percentage of both groups had grade III liver disease. No significant association was found between gender and symptom type across all grades of liver disease (Grade I: χ² = 0.39, p > 0.05; Grade II: χ² = 0.00, p > 0.05; Grade III: χ² = 0.00, p > 0.05). These results indicate that symptom presentation is independent of gender for each liver disease grade (Table 2).

**Table 2:** Patients with typical symptoms and atypical symptoms χ²: Chi-square value

Liver Disease	Typical Symptoms			Atypical Symptoms			χ²	p-value
	Total	Male	Female	Total	Male	Female		
Grade I	34 (46.6%)	12 (35.3%)	22 (64.7%)	14 (43.8%)	7 (50%)	7 (50%)	0.3873	p > 0.05
Grade II	33 (45.2%)	16 (48.5%)	17 (51.5%)	17 (53.1%)	9 (53%)	8 (47%)	0	p > 0.05
Grade III	6 (8.2%)	3 (50%)	3 (50%)	1 (3.1%)	0	1 (100%)	0	p > 0.05

The most common symptom combinations in individuals with liver disease included excessive hunger, urinary frequency, excessive thirst, and evening fatigue, affecting 46 individuals. Other combinations frequently observed involved right upper quadrant pain, thirst, and urinary frequency (Table 3).

**Table 3:** Commonest group of typical symptoms in individuals with liver disease Ayush: Department of Ayurveda, Yoga, and Naturopathy, Unani, Siddha, and Homoeopathy.

Symptom Combination	No of Individuals
Excessive hunger, urinary frequency, excessive thirst, evening fatigue	46
Right upper quadrant pain, urinary frequency	36
Right upper quadrant pain, excessive thirst	33
Right upper quadrant pain, excessive urinary frequency, excessive thirst	32
Right upper quadrant pain, weight loss	14
Right upper quadrant, urinary frequency, excessive thirst, weight loss	13

Individuals presenting after more than two months of symptom onset had a higher prevalence of liver disease (64.3%) and typical symptoms (Table 4). Most of these individuals sought help from MBBS or specialist doctors, while others consulted village doctors or pharmacists. Healthcare consultation varied, with 54.3% of patients consulting MBBS-qualified doctors or specialists and 23.9% seeking help from village doctors. A significant number (33.7%) were referred for specialist care after diabetes detection, primarily by MBBS or specialist doctors (Table 5).

**Table 4:** Distribution of symptoms and liver disease status among individuals with Type 2 diabetes mellitus

	Typical Symptoms of Liver Disease	Typical Symptoms without Liver Disease	Atypical Symptoms with Liver	Disease Atypical Symptoms without Liver Disease	Asymptomatic
Overall	35.9%	18.0%	32.0%	7.7%	6.4%
Individuals presenting after 2 months	64.3%	20.0%	10.0%	5.7%	0.0%

**Table 5:** Healthcare consultation patterns of 92 individuals Ayush: Ayurveda, Yoga, Naturopathy, Unani, Siddha, Homeopathy

Consultation Type	Total Individuals (%)
Consulted Healthcare Professionals	92 (62.2)
1. Village doctors	22 (23.9)
2. Pharmacists	7 (7.6)
3. MBBS-qualified doctors and specialists	50 (54.3)
4. Came directly to this centre	11 (12)
5. Ayush Doctor	2 (2.2)

## Discussion

The high prevalence of liver disease, particularly NAFLD, among individuals newly diagnosed with T2DM underscores the need for comprehensive screening. The significant p-value (0.0124) highlights a strong association between diabetes and liver disease, emphasizing the critical role of early detection. These findings are consistent with Drivsholm et al. (2005), who noted that nine out of ten newly diagnosed persons with Type 2 Diabetes were presented with hyperglycemic symptoms or signs [10,11]. Similarly, Kalra et al. (2013) reported a wide range of NAFLD prevalence (12.5% to 87.5%) in T2DM patients in India [12], supporting the need for focused screening in this population. The presence of typical and atypical symptoms reflects the diverse clinical presentations of T2DM. Typical osmotic symptoms like increased urination and thirst remain predominant, but atypical symptoms, such as lethargy and light-headedness, may delay diagnosis. Educating healthcare providers and patients about these atypical presentations could improve early detection and management, reducing long-term complications. Additionally, asymptomatic individuals accounted for a small portion of the study population, highlighting the need for opportunistic screening in diabetes care. Drivsholm et al. (2005) highlighted that newly diagnosed patients often present with symptoms such as nocturia, polyuria, and weight loss, which are linked to higher glycemic levels [13,14].

Pathway assessment: enhancing early diagnosis and unraveling healthcare delays

Efficient T2DM diagnosis and management rely on a strategic pathway assessment, minimizing resource wastage. Events within the pathway are interconnected, emphasizing the profound impact of earlier decisions on subsequent consequences [15,16]. Three key factors shape outcomes: patient decision-making, clinician choices, and the natural disease progression/treatment response [17]. Understanding these pathways is crucial, delineating associations with care quality, safety practices, resource utilization, and costs. Some pathways signify superior care and safety, while others hint at potential risks [18]. Recognizing these pathways guides interventions to shift patients towards more favorable trajectories, reducing missed opportunities for evidence-based care [19]. The ideal care pathway envisions a patient presenting symptoms to an HCP, leading to a prompt diagnosis, thorough investigations, treatment initiation, and positive patient outcomes.

**Figure 1:**
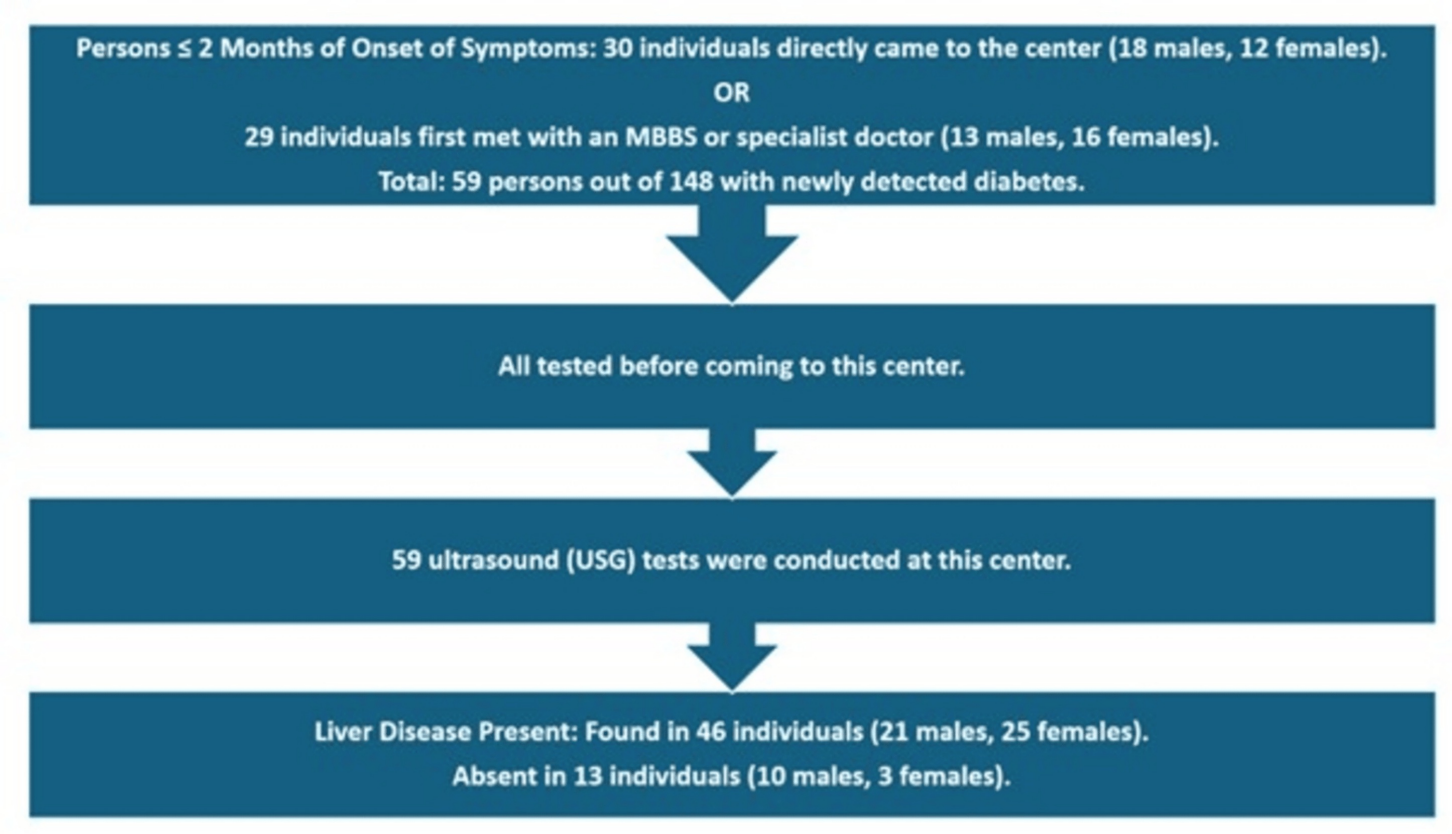
Most efficient path (most favorable path)

Figure 1 shows the most efficient path/most favorable path.

In the most favorable pathway, 143 symptomatic patients were identified, with 73 experiencing symptoms for less than two months. Among these 73 individuals, 59 sought medical help from MBBS-qualified or specialist doctors and were diagnosed with type 2 diabetes mellitus (T2DM) upon testing. Subsequent ultrasound examinations revealed liver diseases in 46 of these patients. Diagnosis occurred within two months of symptom onset, promoting efficient management, appropriate referrals, goal attainment, lifestyle education, and avoidance of unnecessary hospital visits [20]. This pathway highlights the importance of timely intervention by suitable healthcare professionals in managing both T2DM and associated liver diseases (Figure 1).

Less favorable pathway 1

**Figure 2:**
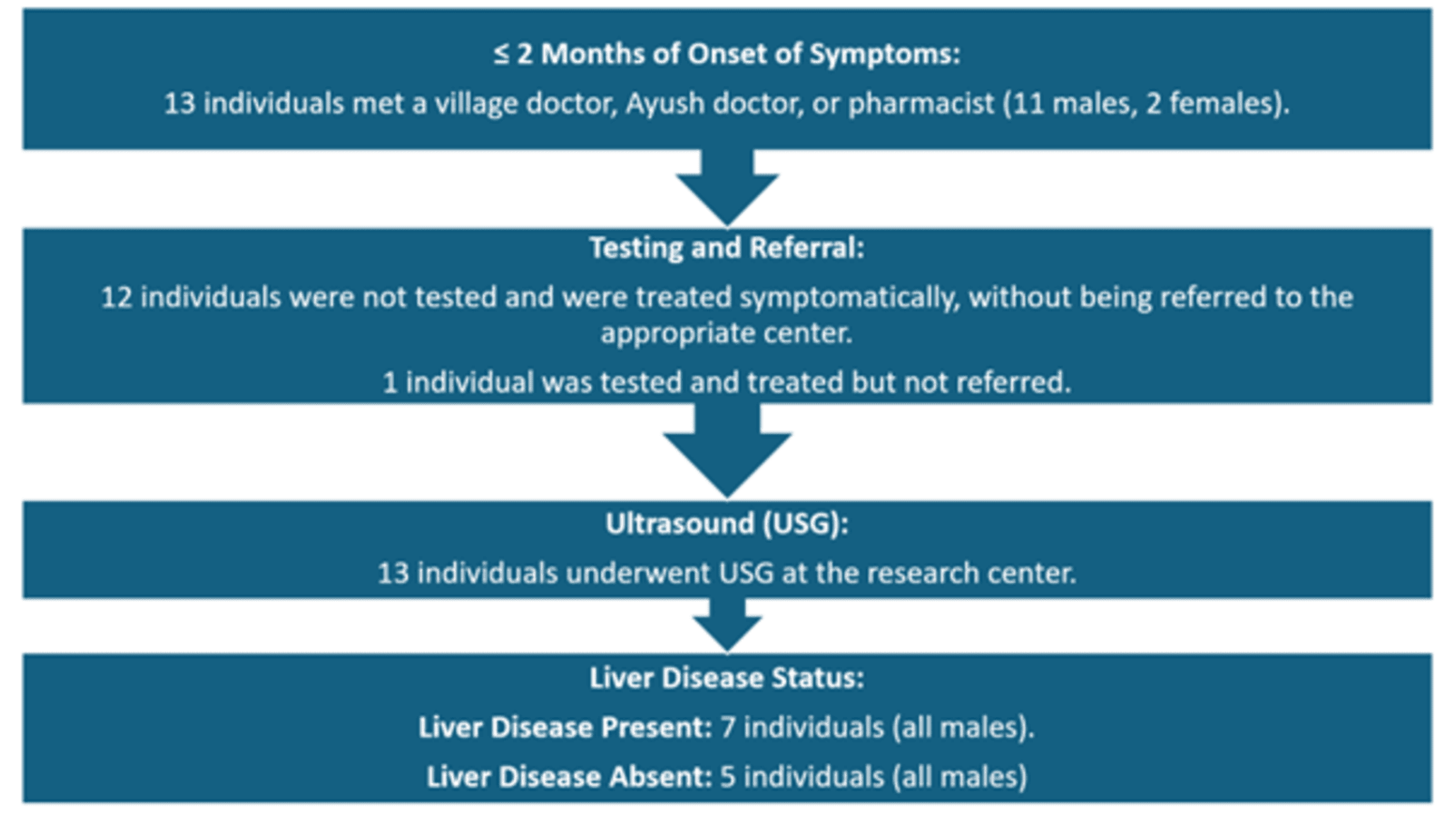
Less favourable pathways

In the less favorable scenario, 13 individuals consulted village doctors, pharmacists, or Ayush doctors. Twelve received only symptomatic treatment without initial testing. Later, they sought testing and ultrasound scans at the center. However, one individual underwent testing but wasn't referred to qualified healthcare professionals. This delay in testing and referral may lead to suboptimal care. Ensuring timely and appropriate medical attention for all seeking healthcare is crucial (Figure 2).

Less favorable pathway 2

**Figure 3:**
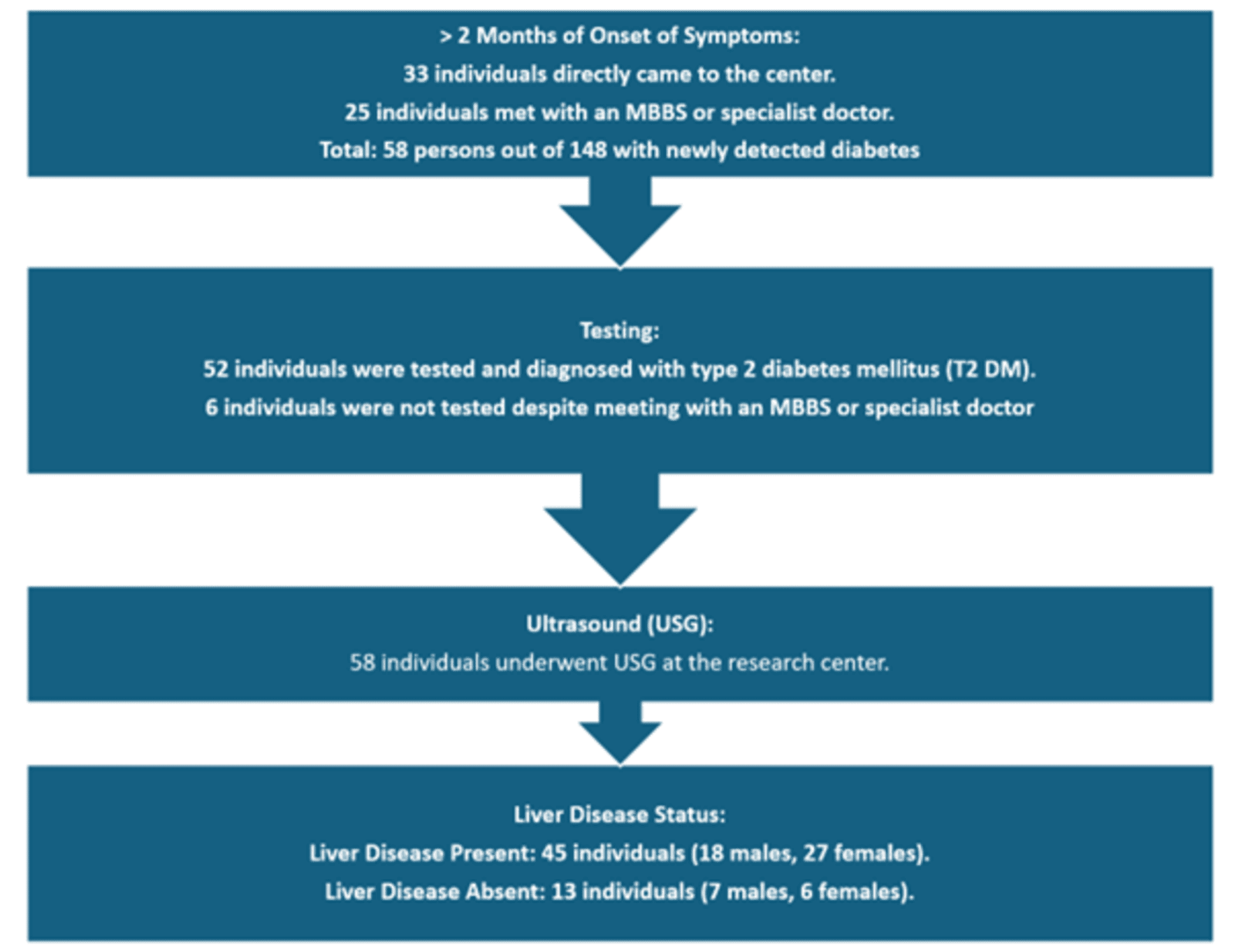
Less favorable pathway

In pathway 2, 58 individuals experienced T2DM symptoms for over two months. Some visited this center or consulted MBBS/specialist doctors, who confirmed the diagnosis. However, six individuals (24%) consulting MBBS/specialist doctors weren't tested, leading to delayed diagnosis. Early detection of T2DM is crucial for timely treatment and complication prevention. Failure to conduct necessary tests may prolong health risks. Upon testing at this center, they were diagnosed with diabetes. Subsequent ultrasounds revealed liver diseases in 45 patients. This highlights the importance of thorough testing by healthcare professionals for prompt diagnosis and management (Figure 3).

Most unfavorable pathway

**Figure 4:**
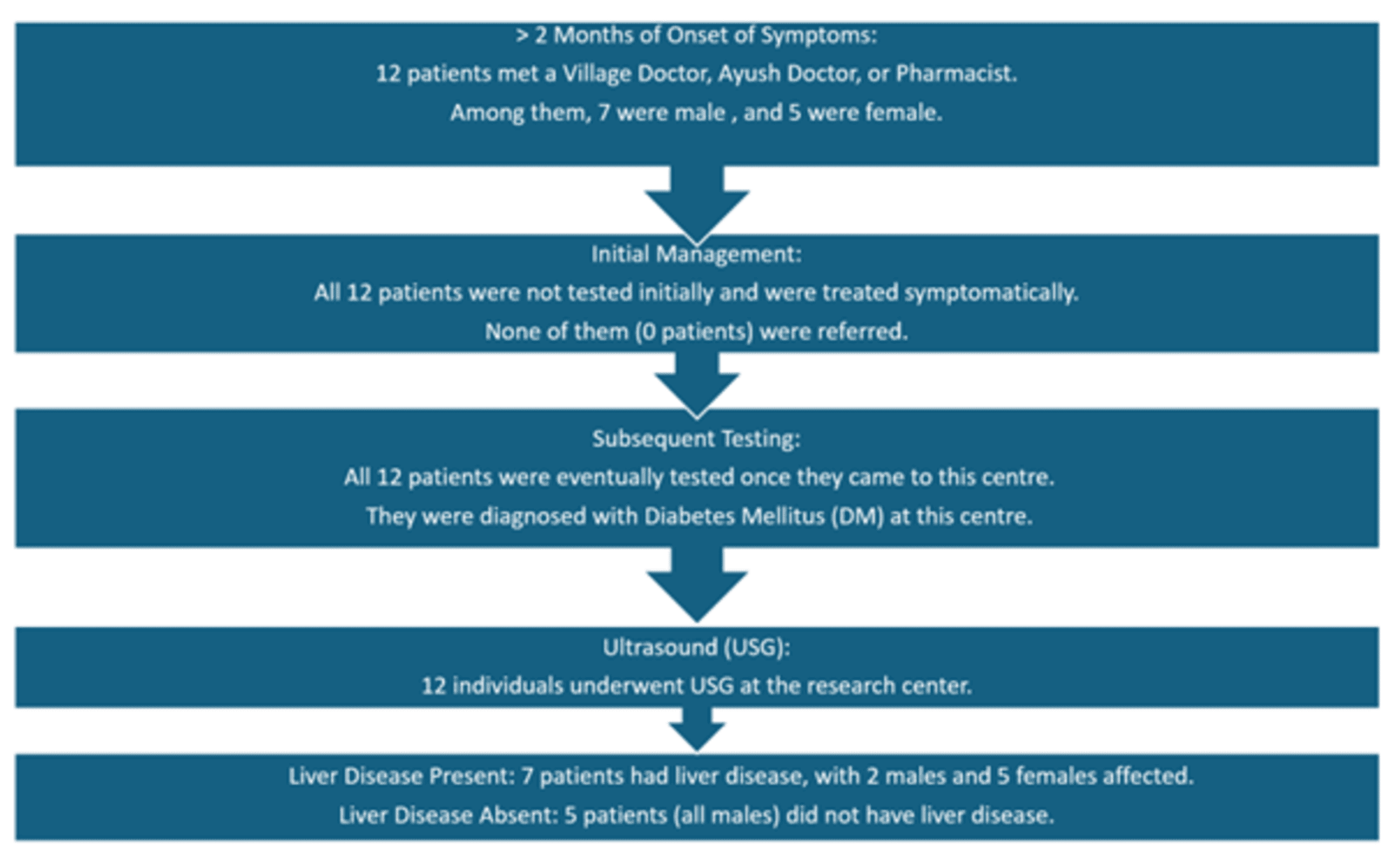
Most unfavorable pathway

In this unfavorable pathway, 12 individuals showed T2DM symptoms over two months. Many visited village doctors, pharmacists, or Ayush doctors who prescribed medications without proper investigation or referrals to specialists. Subsequently, they sought appropriate healthcare once symptoms worsened, causing distress and reduced quality of life. This highlights the urgent need for improved healthcare access and awareness among individuals with diabetes. Proper diagnosis and management by qualified healthcare professionals are crucial. Enhancing primary care providers' capabilities and promoting early intervention can effectively mitigate diabetes-related complications and distress (Figure 4).

Patient factors were identified in this study as contributing to the delays.

In contrast to nations with structured hierarchies, individuals in this region directly decide whom to consult, leading to variations in care quality [21].

Delay in seeking medical attention among T2DM patients for over two months is often due to lack of awareness, symptom recognition difficulties, and prior commitments. Atypical symptoms may further hinder recognition, leading to reliance on local practitioners. Timely education is crucial to promote early intervention. The study highlights missed early T2DM and NAFLD diagnosis opportunities in North Bihar, India. Efficient pathways involving prompt consultation with MBBS or specialist doctors facilitate timely diagnosis, contrasting delays with symptomatic treatment (Table 6).

**Table 6:** Patient factors

Cause of delay > 2 months	
Sr no	Patient factors
1.	Never thought, living with DM
2.	Patient couldn’t relate the symptoms
3.	Atypical symptoms
4.	Did not have time to consult due to work-related engagement
5.	For better care and deteriorating symptoms
6.	Treated by local village doctors and pharmacist
7.	Procrastination

Identifying critical factors

The core challenge in healthcare missed opportunities is the delayed access to knowledgeable healthcare professionals, essential for managing diseases and preventing complications [22,23]. This delay is influenced by various complex factors, which include lack of awareness-based screening to detect asymptomatic stages; limited access to experienced specialists leads to delayed consultations; low health literacy hinders symptom recognition; provider knowledge gaps omit necessary tests and timely referral; competing priorities, the severity of symptoms, and all contribute to shaping this complex journey and cause delays; economic constraints, concepts and cultural beliefs, the idea of symptoms understanding, etiology, and cure further complicate the patient's healthcare trajectory and impede healthcare-seeking behavior [24-26]. These factors collectively contribute to unfavorable pathways, resulting in missed opportunities for early diagnosis and intervention in healthcare.

Limitations

This study has a few limitations, such as cross-sectional design, single-site study, small sample size, and ultrasound basis of diagnosis of liver disease. There may be some variability in imaging and interpretation depending on the sonographer on the findings. However, the same experienced sonographer conducted all the scans, but ideally, the diagnosis of liver disease should be based on liver biopsy. Symptom assessment may be influenced by observer bias. Relying on retrospective data introduces the potential for recall bias, and factors influencing missed opportunities might be multifaceted and not fully captured by the observer.

## Conclusions

The prevalence of NAFLD is notably high among newly diagnosed T2DM cases, influenced by factors such as BMI, lipid profiles, and gender. This emphasizes missed opportunities in healthcare delivery, particularly in the early detection of T2DM, due to delays in accessing knowledgeable professionals and challenges like asymptomatic stages and limited access. The research features the urgent need for future interventions and awareness campaigns to enhance assessment and management. The study offers novel insights into the regional and global understanding of NAFLD and T2DM, particularly through a unique pathway analysis addressing diagnostic delays. Despite acknowledging limitations such as the cross-sectional design and reliance on ultrasound for diagnosis, efforts are made to mitigate these through standardized protocols and comprehensive data collection. The findings hold significant relevance for healthcare policy and practice, especially in resource-limited settings. Future research should prioritize longitudinal studies to validate these findings, explore the temporal relationship between NAFLD and T2DM progression, and integrate advanced diagnostic tools like transient elastography to enhance NAFLD detection accuracy.

## Appendices

Baseline visit 

1. Patient Demographics:

Date of Visit: ____ / ____ / ________ (DD/MM/YYYY) Does the patient satisfy the study selection criteria? □ Yes □ No Patient ID: _______________________ 

Patient Initials: ___________________ Gender: □ Male □ Female

Age: ____________ Years Body Weight: __________Kgs Height: _____________cm.

Body Temperature: ________ o C Pulse Rate: ______________ bpm Respiratory Rate: ________ bpm

2. Physical activity □ Inactive □ Moderately inactive □ Active

3. Comorbidities Sr. no. Comorbidities Yes No Duration (Years / Months) 1. Previous Myocardial Infarction 2. Previous stroke 3. Polycystic ovarian syndrome 4. Obstructive sleep apnoea 5. Vitamin D deficiency 6. Hypothyroidism 7. Family history of type 2 diabetes 8. Family history of liver disease 9. Hypertension (HTN) 10. Dyslipidaemia

4. Investigations

Waist Circumference: _______________ cm Hip Circumference: ______________________cm Waist to Hip ratio: __________________ BMI: ____________________ kg/m2

5.  Laboratory and Diagnostic Investigations

Sr. No. Laboratory Parameter Result 1. FBS _________________(mg/dl) 2. RBS_________________(mg/dl) 3.HbA1c_________________(%) 4. Total Cholesterol_________________(mg/dl) 5. Triglycerides_________________(mg/dl) 6. HDL_________________(mg/dl) 7. LDL_________________(mg/dl) 8. Alanine Transaminase_________________(IU/L) 9. ALT categories Elevated ________________ 10. Ultrasound findings a Grade I b Grade II c Grade III

Data Collection Tool Part B

Date of first review (day/month/year and time): Date of last time patient was well prior to review:

From last time patient was well describe subsequent events:

Date when this happened Description of symptom/event What patient thought

What decision to seek help or not seek help?

If help sought what is outcome. If not seek help, why?
 

Symptoms screening The screening questions below are designed to help populate the table above because it is possible that patients do not know what symptoms are relevant. Therefore, if patients do not volunteer symptoms, then to go through the screening list in the table below and put “X” for “no” and “✔” for “yes”.

Diabetes symptom screen Liver symptoms screen Excessive hunger Urinary frequency Evening fatigue/general fatigue Excessive thirst Involuntary weight gain/loss Itchy private parts Right upper quadrant pain/heaviness Extreme fatigue Generalized itching Blurred vision Jaundice Cut/bruises slow to heal Change in bowel habits Weight loss Leg swelling Tingling/pain/numbness of hand/feet Frequent unexplained infection

13. Have you visited the doctor in the last 6 months? If not, why? For what reason(s)? What treatment did you receive? How did it progress? (This is for screening for other symptoms that might be relevant and not asked). Comments: 

Reference Ranges - 

• Exercise: Inactive, moderately inactive, moderately active, Active As per EPIC-NORFOlK (Cust et al.2008)  

• What was the first symptoms you had? Urination Often, feeling very thirsty, feeling very hungry-even though you are eating, Extreme fatigue, Blurry vision, Cuts/bruises that are slow to heal, Weight loss-even though you are eating more, Tingling, pain, or numbness in the hands/feet, Frequent Unexplained Infections.

(https://www.diabetes.org/diabetes/type-1/symptoms)

Table 3. ULTRASOUND Grading of NAFLD in detected persons

Grade1 Grade II Grade III

Presence of fine diffuse hyper echogenicity is considered mild steatosis. Moderate but diffuse hepatic hyper echogenicity, impaired visualization of intrahepatic vessels and diaphragm was considered moderate steatosis. Marked hepatic hyper echogenicity, borders of intrahepatic vessels, and impaired visualization of diaphragm, and posterior portion of right lobe of liver

Presence of bright liver increased hepatic echotexture than the renal echotexture, presence of vascular blurring and narrowing of hepatic veins were used to detect NAFLD on ultrasound (Joseph et al 1991, Fan et al. 2007).

Investigators Name: ______________________________________

______________________________________________________ Investigator’s Signature and Date Mobile number: _________________________________________ Address _______________________________________________ ______________________________________________________    

Stamp
